# Assessment of Total Phenolic and Flavonoid Content, Antioxidant Properties, and Yield of Aeroponically and Conventionally Grown Leafy Vegetables and Fruit Crops: A Comparative Study

**DOI:** 10.1155/2014/253875

**Published:** 2014-03-23

**Authors:** Suman Chandra, Shabana Khan, Bharathi Avula, Hemant Lata, Min Hye Yang, Mahmoud A. ElSohly, Ikhlas A. Khan

**Affiliations:** ^1^National Center for Natural Products Research, School of Pharmacy, University of Mississippi, University, MS 38677, USA; ^2^Department of Pharmacognosy, School of Pharmacy, University of Mississippi, University, MS 38677, USA; ^3^Department of Pharmaceutics, School of Pharmacy, University of Mississippi, University, MS 38677, USA

## Abstract

A comparison of the product yield, total phenolics, total flavonoids, and antioxidant properties was done in different leafy vegetables/herbs (basil, chard, parsley, and red kale) and fruit crops (bell pepper, cherry tomatoes, cucumber, and squash) grown in aeroponic growing systems (AG) and in the field (FG). An average increase of about 19%, 8%, 65%, 21%, 53%, 35%, 7%, and 50% in the yield was recorded for basil, chard, red kale, parsley, bell pepper, cherry tomatoes, cucumber, and squash, respectively, when grown in aeroponic systems, compared to that grown in the soil. Antioxidant properties of AG and FG crops were evaluated using 2,2-diphenyl-1-picrylhydrazyl (DDPH) and cellular antioxidant (CAA) assays. In general, the study shows that the plants grown in the aeroponic system had a higher yield and comparable phenolics, flavonoids, and antioxidant properties as compared to those grown in the soil.

## 1. Introduction

Over the years, research on antioxidants, as potential therapeutic agents to prevent free radical generated damage in the human body, has gained popularity. Antioxidants of natural origin, compared to the synthetic antioxidants present in the market, have attracted considerable attention by consumers and by researchers since there is concern of synthetic antioxidants consumption due to their instability and possible activity as carcinogens [1–3].

In recent years, consumption of vegetables and fruits in the average diet has been highlighted for its contribution towards lowering the risks of several life-threatening diseases such as coronary heart disease, stroke, pulmonary disease, and different types of cancer [4–13]. The benefits are due to the presence of polyphenols, flavonoids, carotenoids, and vitamins [14–16]. Of these phytochemicals, polyphenols are largely recognized as anti-inflammatory, antiviral, antimicrobial, and antioxidant agents [14].

The concentrations of phenolic and other secondary metabolites in fruits and vegetables are influenced by many factors, including soil, irrigation, and climatic conditions. Soil cultivation of crops may also result in year-to-year variability in the composition of phytochemicals and in total yield [17]. Hence, there is an increased interest in hydroponic/aeroponic cultivation, which has several advantages over traditional soil cultivation including less contact with soil or dust (if grown outdoor). Therefore, there is less chance of contamination through pest and soil-borne pathogens [18, 19]. Furthermore, indoor hydroponic/aeroponic cultivation provides a better control on the quality of the produce in terms of secondary metabolites and crop yield through a complete control on the nutrient supply. Indoor cultivation carried out under the control and optimized environmental conditions can further maximize the yield of the product and also eliminate the problems linked to fluctuations in the outdoor weather conditions.

In the present study, the attempt has been made to evaluate the difference, if any, in quality and quantity of produce between the crops grown in hydroponic/aeroponic systems and in soil.

## 2. Materials and Methods

### 2.1. Plant Material

Seedlings of different leafy vegetables/herbs (basil,* Ocimum basilicum*; chard,* Beta vulgaris*; parsley,* Brassica oleracea*; red kale,* Petroselinum crispum*) and fruit crops (bell pepper,* Capsicum annuum*; cherry tomatoes,* Solanum lycopersicum*; cucumber,* Cucumis sativus*; squash,* Cucurbita pepo*) were grown in 2” jiffy pots during the month of May, 2012. One-month-old, fully developed seedlings were transplanted in the test plot and aeroponic growing systems (Tower Garden by Juice Plus+ aeroponic growing system, Collierville, TN, USA). Seventy-two seedlings of each leafy vegetable and twenty-four seedlings of each fruit crop were planted on 24 Tower Garden aeroponic systems and in the test plot.

The experimental site was located at the field cultivation facility of the National Center for Natural Product Research, Research Institute of Pharmaceutical Sciences, School of Pharmacy, The University of Mississippi. All the Tower Gardens and test plot were kept close to each other to provide similar environmental conditions. Nutrient solution was delivered to each Tower Garden by hand 1-2 times per week as required to keep the volume in the tank between 15 and 20 gallons. The electrical conductivity and pH of nutrient solution in Tower Garden were measured everyday and maintained within the range throughout the experiment. The plants were harvested as the edible produce achieved the earliest harvestable stage. The products of Tower Garden and field grown crops were evaluated and compared for total yield, phenolics, flavonoids, and antioxidant activities using cellular antioxidant (CAA) and 2,2-diphenyl-1-picrylhydrazyl (DDPH) assays.

### 2.2. Measurement of Yield

Fresh weight of each harvest was measured until the final harvest and the total yield was calculated at the end of the season for each crop. Based on the total yield, the number of plants propagated, and the number of fruits produced, the average yield per plant, the average number of fruits per plant, and the average fruit weight were calculated for each crop grown on the Tower Garden aeroponic systems and in the field.

### 2.3. Collection of Samples

Eighteen samples (nine from aeroponic growing systems and nine from the field, 200–400 g each) of each crop were collected for antioxidant analysis, and determination of total phenolic and flavonoids content. Freshly harvested plant material was collected and placed in the container containing dry ice. Immediately after the collection, plant material was brought to the laboratory and stored at −80°C until further use. All the samples were then freeze-dried and ground using a planetary ball mill (PM-400, Retsch, GmbH, Germany) at a low temperature. Out of nine, three randomly selected freeze-dried, powdered samples of each crop from Tower Garden and field were used for further extraction.

### 2.4. Extract Preparation for Total Phenolics, Flavonoids, and Antioxidant Properties

Dried plant material (10 g) from each sample was used for the preparation of extract. Samples were extracted with 75 mL (95% v/v) ethanol at 40°C for 10 min; the extraction process was repeated thrice. The solvent was evaporated at 40°C under a reduced pressure. The dried extract was used for further analysis.

### 2.5. Determination of Total Phenolic and Flavonoids Content

#### 2.5.1. Reagents and Chemicals

Folin-Ciocalteu reagent, gallic acid, and quercetin standards were obtained from Sigma-Aldrich Co. (St Louis, MO, USA). Aluminum chloride hexahydrate, methanol, and sodium carbonate were obtained from Fisher Scientific (Fair Lawn, NJ, USA). Water was purified using a Milli-Q system (Millipore).

#### 2.5.2. Sample Preparation

About 10–50 mg of the extract was dissolved in 5 mL methanol and sonicated for 45 minutes at 40°C followed by centrifugation at 1,000 ×g for 10 min. The clear supernatant was collected and stored in an amber bottle for analysis.

#### 2.5.3. Total Phenolic Content

The total phenolics of the extracts were determined using the Folin and Ciocalteu reagent, following the method described by Singleton and Rossi [20] with slight modifications. Sample and standard readings were made using a spectrophotometer (Cary 50 Bio UV-Vis Spectrophotometer, Varian) at 765 nm against the reagent blank.

The test sample (0.2 mL) was mixed with 0.6 mL of water and 0.2 mL of Folin-Ciocalteu's phenol reagent (1 : 1). After 5 min, 1 mL of saturated sodium carbonate solution (8% w/v in water) was added to the mixture and the volume was made up to 3 mL with distilled water. The reaction was kept in the dark for 30 min and after centrifuging the absorbance of blue color from different samples was measured at 765 nm. The phenolic content was calculated as gallic acid equivalents GAE/g of dry plant material on the basis of a standard curve of gallic acid (5–500 mg/L, *Y* = 0.0027*x* − 0.0055, *R* ^2^ = 0.9999). All determinations were carried out in triplicate.

#### 2.5.4. Total Flavonoids Content

The aluminum chloride colorimetric method was used for the determination of the total flavonoid content of the sample [21–24]. For total flavonoid determination, quercetin was used to make the standard calibration curve. Stock quercetin solution was prepared by dissolving 5.0 mg quercetin in 1.0 mL methanol, then the standard solutions of quercetin were prepared by serial dilutions using methanol (5–200 *μ*g/mL). An amount of 0.6 mL diluted standard quercetin solutions or extracts was separately mixed with 0.6 mL of 2% aluminum chloride. After mixing, the solution was incubated for 60 min at room temperature. The absorbance of the reaction mixtures was measured against blank at 420 nm wavelength with a Varian UV-Vis spectrophotometer (Cary 50 Bio UV-Vis Spectrophotometer, Varian). The concentration of total flavonoid content in the test samples was calculated from the calibration plot (*Y* = 0.0162*x* + 0.0044, *R* ^2^ = 0.999) and expressed as mg quercetin equivalent (QE)/g of dried plant material. All the determinations were carried out in triplicate.

### 2.6. Determination of Antioxidant Activity

The extracts were dissolved in dimethyl sulfoxide (DMSO) to make a stock solution of 20 mg/mL. The antioxidant activity of the extracts was measured at a concentration of 500 *μ*g/mL by following two methods.

#### 2.6.1. 2,2-Diphenyl-1-picrylhydrazyl (DPPH) Assay

The capacity of plant extracts (500 *μ*g/mL) to directly react with and quench free radicals was evaluated as described earlier [25]. A stock solution of DPPH (200 *μ*M) was prepared in ethanol. The assay was performed in 96-well plates. The reaction mixture, containing 100 *μ*L of DPPH and 100 *μ*L of the diluted test sample, was incubated at 37°C for 30 min. The absorbance was measured at 515 nm. Gallic acid was used as a positive control. Percent DPPH radical scavenging activity was calculated as follows:

(1) Percent  radical  scavenging  activity  ={1−(sample−blank)(control−blank)}×100.

Gallic acid showed 95% radical scavenging activity at 20 *μ*M.

#### 2.6.2. Cellular Antioxidant Activity Assay (CAA Assay)

The cellular antioxidant activity was measured in HepG_2_ cells as described by Wolfe and Rui [26]. The method measures the ability of phytochemicals in the plant extracts to prevent intracellular generation of peroxy radicals in response to ABAP (used as a generator of peroxyl radicals). The CAA assay is a more biologically relevant method than a chemical assay because it represents the complexity of biological system and accounts for cellular uptake, bioavailability, and metabolism of the antioxidant agent.

HepG_2_ cells (acquired from American type culture collection, ATTC, Rockville, MD) were grown in DMEM supplemented with 10% FBS and antibiotics (50 units/mL penicillin and 50 *μ*g/mL streptomycin). For the assay, cells were seeded in the wells of a 96-well plate at a density of 60,000 cells/well and incubated for 24 hrs. The medium was removed and cells were washed with PBS before treating with the test sample (500 *μ*g/mL) diluted in the medium containing 25 *μ*M DCFH-DA for 1 hr. After removing the medium, the cells were treated with 600 *μ*M ABAP and the plate was immediately placed on a SpectraMax plate reader for kinetic measurement every 5 min for 1 hr (37°C, emission at 538 and excitation at 485 nm). Quercetin was used as the positive control. The antioxidant activity was expressed in terms of CAA units. The area under the curve (AUC) of fluorescence versus time plot was used to calculate CAA units as described by Wolfe and Rui [26]:

(2) $$\begin{array}{c} \text{CAA}\;\;\text{unit}=100-\left\{\left(\frac{\text{AUC}\;\;\text{sample}}{\text{AUC}\;\;\text{control}}\;\right)\times 100\right\}. \end{array}$$

Quercetin showed CAA unit of 60 at 16 *μ*M. This indicates that quercetin (at 16 *μ*M) caused 60% inhibition of cellular generation of peroxyl radicals in HepG_2_ cells.

### 2.7. Statistical Analysis

All the experiments for determination of total phenolics, total flavonoids, and antioxidant properties using DPPH and cellular antioxidant assay (CAA) were conducted in triplicates. The values are expressed as the mean ± standard deviation (SD). Average crop yield was calculated by dividing total yield by number of plants grown. The statistical analysis of the results was done by agricolae module using *R*-statistical software package version 2.2.1 (*R* foundation for statistical computing, Vienna, Austria) [27]. Analysis of variance and significance of difference among means were tested by one-way ANOVA and least significant difference (LSD) on mean values. Correlation coefficients (*r*) and coefficients of determination (*r* ^2^) were calculated using Microsoft Excel 2007.

## 3. Results

### 3.1. Crops Yield

Table 1 shows the comparison in the average crop yield per plant, average fruit weight, and average number of fruits per plant in different crops grown in aeroponic growing systems (AG) and in the field (FG). The average crop yield per plant (and total yield) was higher in the crops grown in aeroponic systems as compared to those grown in the field. An average increase of about 19%, 8%, 65%, and 21% in yield was recorded in basil, chard, red kale, and parsley (leafy vegetables) when grown in aeroponic systems. Similarly, an average increase of about 53%, 35%, 7%, and 50% in yield was recorded in bell pepper, cherry tomatoes, cucumber, and squash (fruit crops), respectively, when grown in aeroponic systems as compared to those plants grown in the soil. The average weight of the cucumbers was higher in field grown plants, whereas the average weight of squash and bell peppers was higher in the plants grown in aeroponic systems. A comparable average fruit weight (21.78 g in FG and 20.61 g in AG) was observed for cherry tomatoes grown in the two types of growing systems. On the other hand, the average number of fruit produced per plant was higher in all the fruit crops grown in the Tower Garden aeroponic systems as compared to those grown in soil. 

### 3.2. Determination of Total Phenolic Content

**Figure 1:**
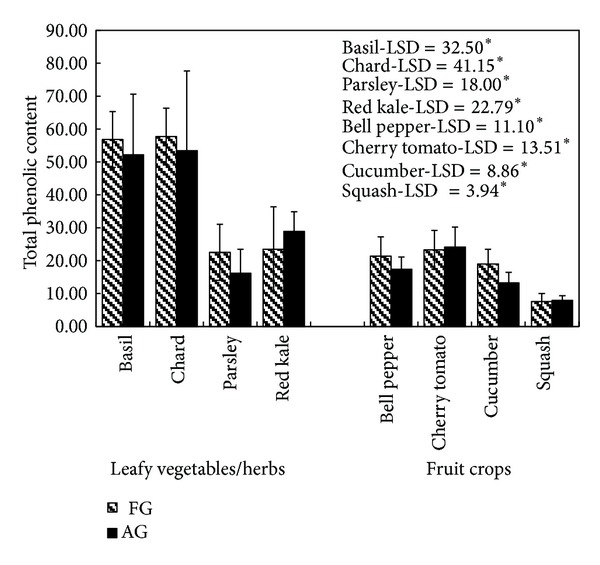
A comparison of total phenolic content (mg GAE/g dry wt.) in different crops grown in the field (FG) and in aeroponic growing systems (AG); data represent mean ± SD, *n* = 3; LSD: least significant difference; level of significance: *P < 0.05.

Figure 1 shows the total phenolic content in the samples of different leafy vegetable and fruit crops grown in Tower Garden aeroponic systems and in the soil. Among the leafy vegetables, the highest phenolic content was found in chard (57.73 mg GAE/g dry wt., in FG and 53.45 GAE/g dry weight in AG) followed by basil, red kale, and parsley. Phenolic content was slightly higher in basil, chard, and parsley when grown in soil as compared to those grown in aeroponic systems, whereas phenolic content was slightly higher in aeroponically grown red kale as compared to those grown in the soil. The differences in phenolic content, however, were not found to be statistically significant for all the leafy vegetables (basil, LSD = 32.50, *P* < 0.05; chard, LSD = 41.15, *P* < 0.05; parsley, LSD = 18.00, *P* < 0.05; red kale, LSD = 22.79, *P* < 0.05) while grown in two types of cultivation systems. Similarly, differences in phenolic content in aeroponically and field grown fruit crops bell pepper (LSD = 11.10, *P* < 0.05), cherry tomatoes (LSD = 13.51, *P* < 0.05), cucumber (LSD = 8.86, *P* < 0.05), and squash (LSD = 3.94, *P* < 0.05) were also observed to be statistically insignificant. Leafy vegetables, in general, have shown higher phenolic content as compared to fruit crops irrespective of cultivation systems.

### 3.3. Determination of Flavonoids Content

**Figure 2:**
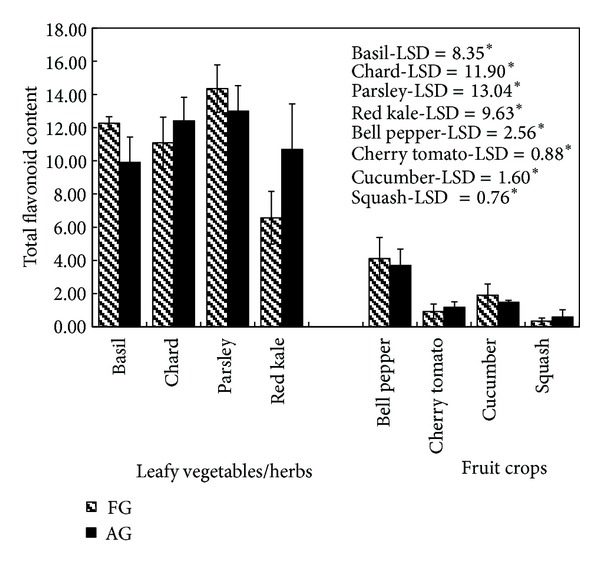
A comparison of total flavonoid content (mg QE/g dry wt.) in different crops grown in the field (FG) and aeroponic growing systems (AG); data represent mean ± SD, *n* = 3; LSD: least significant difference; level of significance: *P < 0.05.

The total flavonoids content in different crops grown in aeroponic systems and in the field are shown in Figure 2. Among leafy vegetables, the highest amount of flavonoid content was found in parsley (14.35 mg quercetin acid equivalent (QE)/g dry wt. in FG and 13.00 QE/g dry weight in AG) followed by chard (11.08 mg QE/g dry wt. in FG and 12.41 QE/g dry wt. in AG), basil (12.27 mg QE/g in FG and 9.91 QE/g dry wt. in AG), and red kale (6.57 mg QE/g dry wt. in FG and 10.69 QE/g dry wt. in AG), whereas in fruit crops, the flavonoid content was the highest in bell pepper (4.11 mg QE/g dry wt. in FG and 3.70 QE/g dry wt. in AG) followed by cucumber, tomato, and squash. The differences in flavonoid content in aeroponic and field grown crops, basil (LSD = 8.35, *P* < 0.05), chard (LSD = 11.19, *P* < 0.05), parsley (LSD = 13.04, *P* < 0.05), red kale (LSD = 9.63, *P* < 0.05), bell pepper (LSD = 2.56, *P* < 0.05), cherry tomatoes (LSD = 0.88, *P* < 0.05), cucumber (LSD = 1.60, *P* < 0.05), and squash (LSD = 0.76, *P* < 0.05) were observed to be statistically insignificant. Similar to phenolic content leafy vegetables had higher flavonoid content compared to fruit crops.

### 3.4. Determination of Antioxidant Activity

#### 3.4.1. 2,2-Diphenyl-1-picrylhydrazyl (DPPH) Assay

**Figure 3:**
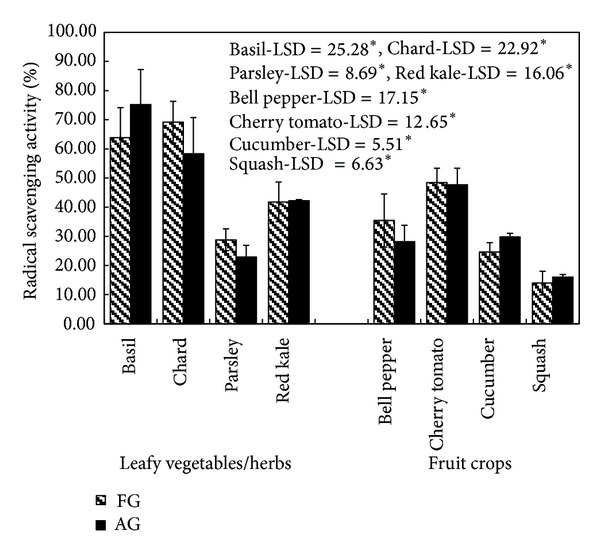
Antioxidant activity of field grown (FG) and aeroponic grown (AG) plants at 500 *μ*g/mL by DPPH assay; data represent mean ± SD, *n* = 3; LSD: least significant difference; level of significance: *P < 0.05.

Antioxidant properties of AG and FG crops using 2,2-diphenyl-1-picrylhydrazyl (DDPH) assay are shown in Figure 3. Among leafy vegetables, antioxidant activity in terms of radical scavenging activity, using DPPH assay, ranged between 63.88 and 28.80% in the field grown crops, whereas it ranged between 75.22 and 22.91% in the plants grown in aeroponic systems. The maximum antioxidant activity was observed in basil and the minimum in parsley among the leafy vegetables. In general, values of radical scavenging activity were lower in fruit crops as compared to those of leafy vegetables. Among the fruit crops, the activity ranged between 48.47 and 13.93% in field grown crops, whereas it ranged between 47.70 and 16.01% in the crops grown in aeroponic systems. The maximum activity was found in cherry tomatoes and the minimum activity was observed in squash among fruit crops. Radical scavenging activity of aeroponically grown crops was found comparable to those grown in the field. The minor differences in the radical scavenging activity between aeroponically grown crops and those grown in soil were, however, statistically insignificant (basil, LSD = 25.28; chard, LSD = 22.92; parsley, LSD = 8.69; red kale, LSD = 16.06; bell pepper, LSD = 17.15; cherry tomatoes, LSD = 12.65; cucumber, LSD = 5.51; squash, LSD = 6.63) (*P* < 0.05).

#### 3.4.2. Cellular Antioxidant Assay (CAA)

**Figure 4:**
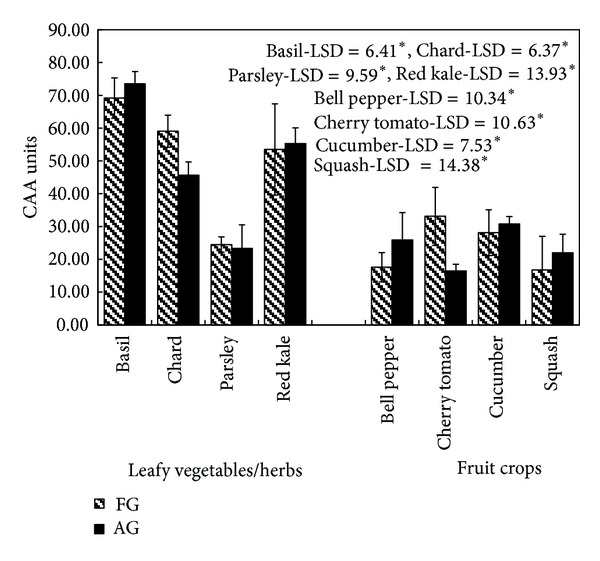
Antioxidant activity of field grown (FG) and aeroponic grown (AG) plants at 500 *μ*g/mL by cellular antioxidant assay (CAA); data represent mean ± SD, *n* = 3; LSD: least significant difference; level of significance: *P < 0.05.

Antioxidant activities of AG and FG crops using the cellular antioxidant assay (CAA) are shown in Figure 4. Similar to DPPH assay, the maximum antioxidant activity, among the leafy vegetables, was found in basil (69.18 CAA units in FG and 73.52 CAA units in AG) and minimum was in parsley (24.51 CAA units in FG and 23.33 CAA units in AG), whereas among fruit crops, maximum activity was in field grown cherry tomatoes (33.11 CAA units) and minimum in field grown squash (14.38 CAA units). Except for tomatoes and chard, all other crops had comparable antioxidant activity (*P* < 0.05) as determined by CAA assay. The activity of tomatoes (LSD = 10.63, *P* < 0.05 and LSD = 15.46, *P* < 0.01) and chard (LSD = 6.37, *P* < 0.05 and LSD = 9.05, *P* < 0.01) was higher in field grown plants as compared to those grown in aeroponic systems.

## 4. Discussion

Plants are potential sources of natural antioxidants. Fruits and vegetables in the diet have been shown in epidemiological studies to be protective against several chronic diseases associated with aging such as cancer, cardiovascular disease, cataracts, and brain and immune dysfunction [28–30]. These natural protective effects have been attributed to various components such as carotenoids, vitamins C and E, and phenolic and thiol (SH) compounds [31]. Many studies have focused on the biological activities of phenolics which are potent antioxidants and free radical scavengers [32–34]. The antioxidant activity of phenolics is mainly due to their redox properties, which allows them to act as reducing agents, hydrogen donors, and singlet oxygen quenchers [33, 35, 36]. The interest in phenolic compounds derived from vegetables and their roles in nutrition are therefore increasing [32, 37]. Phenolic compounds are also known to play an important role in stabilizing lipids against peroxidation and inhibiting various types of oxidizing enzymes [38, 39]. The differences in the flavonoid structures and their substitutions influence the phenoxyl radical stability, thereby affecting the antioxidant properties of the flavonoids [40]. In the present study, phenolics and flavonoid content of aeroponically grown crops were found to be comparable to those grown in the soil. The total product yield was, however, higher in aeroponically grown crops. In a similar study, Miller et al. (1989) [41] have reported a greater dry-matter accumulation in maize (*Zea mays* L.) on the hydroponic system than in well-fertilized, irrigated sandy-loam soil when planting pattern and density were the same. In another study, a significantly higher phenolic content in basil leaves has been reported by Sgherri et al. (2010) [42] while grown in hydroponics as compared to those grown in soil.

The antioxidant capacity of fruits and vegetables can be tested using a wide variety of methods. In the present study, the antioxidant activity of the fresh produce was evaluated in terms of their free radical scavenging capacity by DPPH assay. Their activity against intracellular oxidative stress was determined by CAA assay. These assays have frequently been used by researchers to assess antioxidant capacity of different food products [43–45]. Our results show that the radical scavenging activity of aeroponically grown crops was highly comparable to those grown in the field. Sgherri et al. (2010), using similar methods, have reported an improved antioxidant activity of both aqueous and lipid extracts of basil leaves in hydroponic cultivation as compared to those grown in soil.

**Figure 5:**
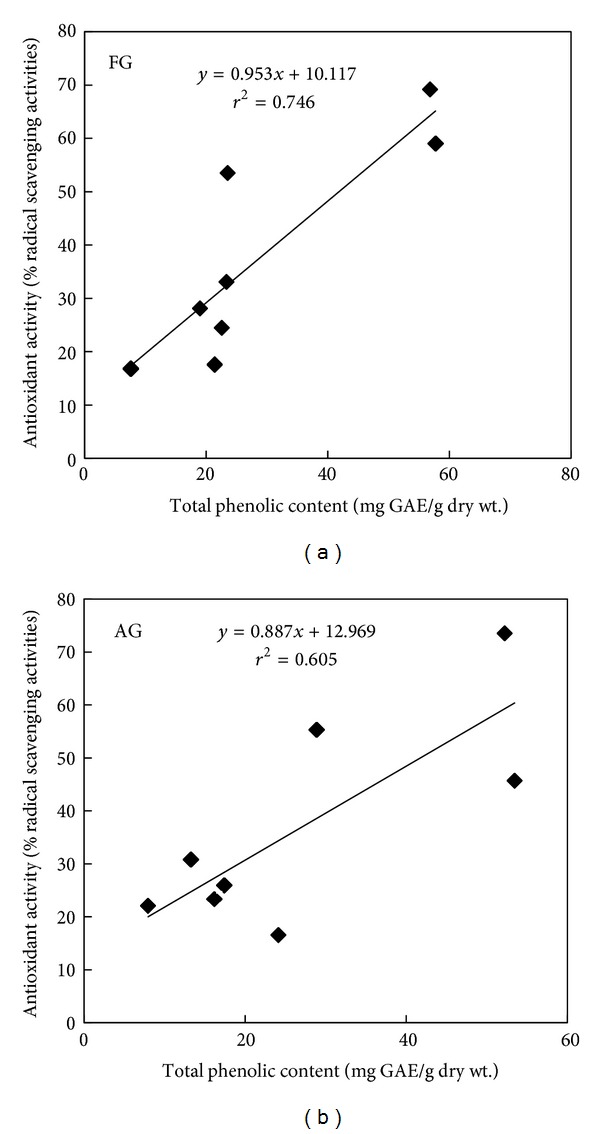
Relationship between total phenolic content and antioxidant activity of field grown (FG) and aeroponic grown (AG) plants by DPPH assay.

**Figure 6:**
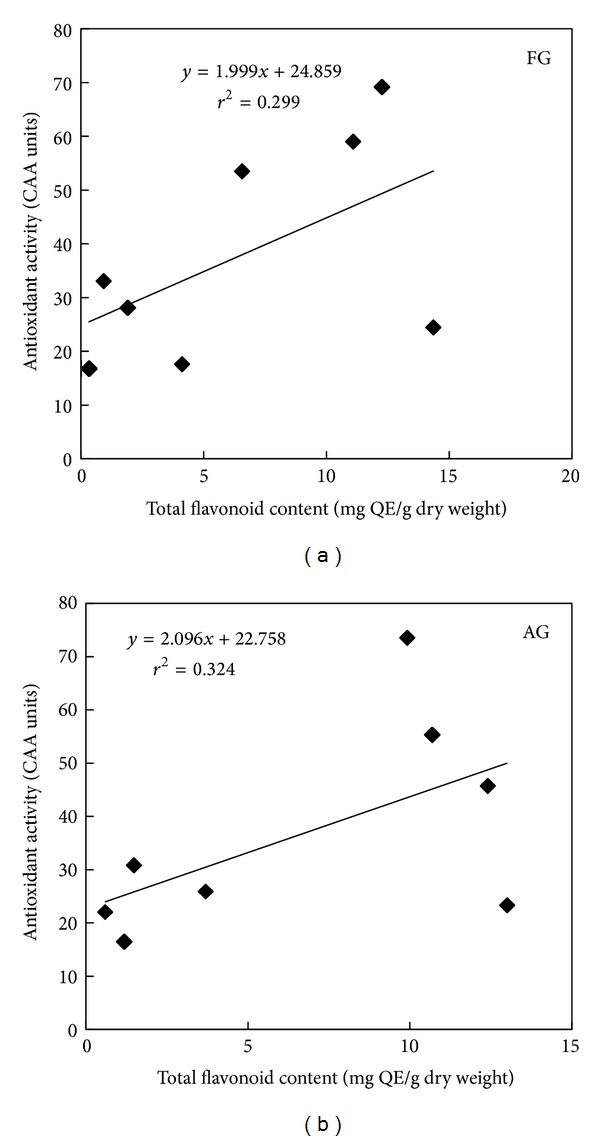
Relationship between total flavonoid content and antioxidant activity of field grown (FG) and aeroponic grown (AG) plants by cellular antioxidant assay.

The relationship between total phenolic content and antioxidant activity using DPPH assay and total flavonoid and antioxidant activity using cellular antioxidant assay in different crops grown in aeroponic systems and in the field is shown in Figures 5 and 6, respectively. Following DPPH assay, regression analysis shows that phenolic compounds contribute to about 75% (*r* ^2^ = 0.746, *P* < 0.05) and 61% (*r* ^2^ = 0.605, *P* < 0.05) of radical scavenging properties in the crops grown in field and in Tower Garden, respectively (Figure 5). Similarly, flavonoids contribute to about 30% (*r* ^2^ = 0.299, *P* < 0.05) and 32% (*r* ^2^ = 0.324, *P* < 0.05) of antioxidant activity in the crops grown in the field and in Tower Garden, respectively (Figure 6). Evidently, the rest of the proportion of antioxidant activity comes from nonphenolic compounds such as vitamins and carotenoids [46]. Phenolics and flavonoids, in general, constitute a major group of compounds, which act as primary antioxidants [47], and are known to react with hydroxyl radicals [48], superoxide anion radicals [49], and lipid peroxy radicals [50]. They are also known to protect DNA from oxidative damage, inhibit growth of tumor cells and possess anti-inflammatory and antimicrobial properties. Similarly, Yao et al. 2010 [51] reported a significant positive correlation between the antioxidant activity and the contents of total flavonoids and total phenolics in celery. The higher proportion of antioxidant activity of phenolic compounds in the species grown in aeroponic systems and in the soil in our study can be used as an accessible source of natural antioxidants. Our data suggests that in spite of a few variations, antioxidant activities of aeroponically grown crops were highly comparable to those grown in soil. Since concentrations of vitamins and phenolic compounds in the crop produce may be influenced by uneven distribution of nutrients in the soil, the hydroponic/aeroponic systems provide a higher level of reproducibility, which is a prerequisite if the product is being used in the food or nutraceutical industry.

In conclusion, the study reveals that plants grown in aeroponic systems show a higher product yield and comparable antioxidant properties (using DPPH and cell-based assays) to those grown in the soil.

## Figures and Tables

**Table 1:** A comparison of average yield in different leafy vegetables (*n* = 72) and fruit crops (*n* = 24) grown in the field and aeroponics systems. FG: field grown plants, AG: aeroponic grown plants.

Plant species	Average yield per plant (g)		Average fruit weight (g)		Average number of fruits produced per plant	
	FG	AG	FG	AG	FG	AG
Leafy greens						
Basil (*Ocimum basilicum*)	326.64	388.14	NA	NA	NA	NA
Chard (*Beta vulgaris*)	228.22	246.78	NA	NA	NA	NA
Parsley (*Petroselinum crispum*)	342.04	414.64	NA	NA	NA	NA
Red kale (*Brassica oleracea*)	272.56	450.24	NA	NA	NA	NA

Fruit crops						
Bell pepper (*Capsicum annuum*)	834.54	1277.88	92.73	116.17	9.00	11.00
Cherry tomato (*Solanum lycopersicum*)	3513.58	4741.83	21.78	20.61	161.00	230.00
Cucumber (*Cucumis sativus*)	4427.38	4727.38	316.24	225.11	14.00	21.00
Squash (*Cucurbita pepo*)	836.17	1249.92	167.23	208.32	5.00	6.00

NA: not applicable.
